# Application of the Second Law of Thermodynamics in Brazilian Residential Appliances towards a Rational Use of Energy

**DOI:** 10.3390/e22060616

**Published:** 2020-06-02

**Authors:** Carlos Eduardo Keutenedjian Mady, Clara Reis Pinto, Marina Torelli Reis Martins Pereira

**Affiliations:** School of Mechanical Engineering, University of Campinas, Mendeleyev St., 200—Cidade Universitária, Campinas 13083-970, Brazil; clara.reis.p@gmail.com (C.R.P.); torellimarina@gmail.com (M.T.R.M.P.)

**Keywords:** exergy analysis, exergy efficiency, energy labelling program, residential appliances, electric shower, air conditioning

## Abstract

This article proposes the utilization of the concepts of destroyed exergy and exergy efficiency for equipment and process performance indicators that are related to the current energy planning scenario in Brazil, more specifically with energy-efficiency labelling. Several indicators associated with these concepts are discussed, including one national program that is based on labeling the energy efficiency of several residential, commercial and industrial appliances. The grades are indicated in the equipment using values from A to G. This labeling system is useful for discriminating similar technologies used for the same function; nevertheless produced by different enterprises. For this complementary analysis, two types of refrigeration methods were compared, absorption and vapor compression; however, these energy indexes alone are not sufficient parameters to select among these two technologies, because their performance indexes definition are different. To address this, our research considers the second law of thermodynamics through exergy analysis as a proper sub-index to obtain a systematic comparison between these various indicators. It is significant to highlight that seldom research studies addressed to this problem so explicitly, in an actual governmental working solution, aiming at discussing to the society the advantage of the usage of the “quality of the energy” as a complementary index to governmental and personal choices. Results indicate that it is possible to use the destroyed exergy and exergy efficiency to help select the technology that better utilizes natural resources, considering the energy matrix of the country. Appliances for water heating and air conditioning were compared from energy and exergy viewpoint, where the last gave additional information about the quality of energy conversion process, giving a completely different trend from the energy analysis alone, without the necessity to think about the energy matrix. Later this issue is addressed from both points of view. Future studies may suggest an exergy based index. The energy efficiency suggests that electrical shower (values higher than 95%) are better than gas water heaters (83%) in using natural resources, whereas the exergy efficiency shares similar magnitudes (about 3%). A related pattern is shown for the theoretical air conditioning systems. The vapor compression systems have an energy index higher than 3, and absorption systems lower than 1. For these circumstances, the exergy efficiency shows figures nearby 30%.

## 1. Introduction

The first law of thermodynamics states that there is conservation in every energy conversion process, meaning that there is neither a generation nor destruction in the total amount of energy. However, nothing is stated in the law regarding the quality (nobility) of energy. Although the quest for an analytical formulation for the maximum amount of work has a long history in literature, the term exergy, first proposed by Rant [1], is defined as the maximum possible work for a given energy transfer [2], interacting only with the environment (known parameter) [3]. Several authors have evaluated the applicability of the second law of thermodynamics as a decision index to improve the quality of the energy conversion process. The exergy analysis is a proper tool to evaluate different processes, ranging from biological systems [4] to societies [5]. Therefore, it is possible to evaluate the vestiges lost within the environment by any given process [6].

In the past decades, the concept has been widely used by public and private institutions [7,8] to evaluate the efficiency of energy use of systems in various sectors, including industrial [9,10], transport [11], agriculture [12], commercial/services [13], and residential [14].

Similar to energy analysis, exergy analysis is a valuable tool for improving the efficiency of energy conversion processes and contributing in decision-making by identifying areas where there are higher losses of work capability or desired end product. One of the first attempts was made by Neves and Seader [15], in which the lost work was used to evaluate efficiency parameters. Moreover, some studies used the exergy analysis to propose a different class of energy taxation [16].

Further, the inclusion of exergy analysis in decision-making in energy planning offers advantages that go beyond the information obtained through the so-called “energy balances” [17]. These include (i) the identification of thermodynamic limitations [17,18,19]; (ii) the replacement of technologies in complex systems [17]; (iii) the location and magnitude of the degradation of the quality of the energy (resulting from heat transfer, reaction, or other conversion process) [17]; (iv) the adequacy between the quality of the supplied and demanded energy [17]; and (v) information for the environmental impact analysis [17]

Thus, in the study of energy planning, the evaluating of the life cycle of energy-intensive products and processes combined with their exergy analysis is of great interest. This application type consists of using exergy as a standard reference unit in a product life cycle assessment. The known assessment methods of this type in the literature include the Life Cycle Exergy Analysis (LCEA) proposed by Wall and Gong [18], Ref. [19] and the similar Exergetic Life Cycle Analysis (ELCA) submitted by Cornelissen [20], which focuses on exergy loss during the complete life cycle of a product [21]. There is also the cumulative exergy consumption indicator (CExC) developed by Szargut et al. [2]. Recently, the use of parameters such as the renewable energy index has gained attention [6]; however, there is currently no consensus in literature regarding an exergy index.

Over the past decades, there has been a systematic effort of the Brazilian governments to achieve a rational use of electrical energy for residential appliances [22,23]. Programs such as the “INMETRO Brazilian Labeling Program” [24,25], have been developed and continuously modified over time. This effort is a successful example of how simple information given to the consumer helps increasing the quality of the energy conversion in the residential sector (from grade G to A). Moreover, if all consumers were to have used, for instance, air conditioning with the top efficiency label of A, it might have been possible to achieve annual savings of 322 GWh of energy in 2007 [25].

As discussed by Vendruscolo [24], using electricity to heat water or to heat and cool air for refrigerators and air conditioning may account for more than 60% of the end-use of energy in Brazil. Other appliances usually found are electrical oven, electric cooktop, microwaves, lights, wash-machines [26]. With this figure, it is essential to consider the second law of thermodynamics since the energy conversion from a source of high quality (electricity) to a grade with lower quality (heat transfer at 45–60 °C) is irreversible [27].

The energy analysis may be sufficient when there is a comparison between same types of equipment with the same energy input (air conditioning with vapor compression). Nevertheless, when there is a comparison of absorption chillers with refrigeration systems using vapor compression, there is difficulty in utilizing the performance indicator (COP), since the nature of the energy input is different. Other home appliances may also be used as examples, such as electric showers, gas water heaters, or solar panels [6].

In this article, the application of the destroyed exergy and exergy efficiency are proposed as indexes, complementary to energy efficiency or other based energy indexes. These energy based thermodynamic indicators are extensively used in Brazil [28], e.g., in the “INMETRO Brazilian Labeling Program”. These energy indexes may be used in multiple applications from electric showers to buildings [24,25]. It is not the purpose of this article to criticize the First Law of Thermodynamics based indexes. Moreover, the exergy analysis is proposed to be a complementary indicator, since it is not yet generally well-known to the final consumer or to engineers. It is in the authors’ knowledge that most consumers do not buy a residential appliance based on CO${}_{2}$ emissions nor a reduction in the monthly payments to the electrical company aiming a longterm payback. Nevertheless, as indicated by [15,16] it would be a purpose of the policymakers to make taxation decisions based not solely on the First Law of Thermodynamics, but also aiming the rational use of energy. This procedure may stimulate the driving factors decision making for each end costumers.

Two types of energy conversion processes are analyzed herein: water heaters and air-conditioning. A general application of this exergy analysis is offered as a basis for identification and indexation that allows end-customers to select which technology to apply to their residence. Moreover, this article intends to bring this newly modified index to the attention of policymakers in order to utilize in the best possible way the several available indexes of public policy and encourage a more careful use of energy in order to achieve the United Nations’ sustainable development goals [29].

## 2. Methods

According to De Melo and Jannuzzi [30], power performance standards begin with the “energy efficiency act” and this was adopted for cooling systems in 2007 [26]. Based on this, two examples of energy use were analyzed from the viewpoints of the first and second laws of thermodynamics for the Brazilian scenario. Firstly, a comparison of the indexes used in literature was carried out to evaluate these cycles (COP and exergy efficiency). Moreover, as stated by Vendrusculo et al. [24], various methods of heating water by means of electrical energy account for 30% of the energy consumption of the residential sector. These technologies are labeled from the perspective of the first law of thermodynamics. All equipment is required to have these labels in order to be commercialized.

The first law of thermodynamics for a general control volume (CV) can be obtained from Equation (1) disregarding the variation of potential and kinetic energy.

(1) $$\frac{dU_{CV}}{dt}=\sum {\dot{m}}_{e}h_{e}\sum {\dot{m}}_{s}h_{s}+{\dot{Q}}_{CV}-{\dot{W}}_{CV}$$

where *U* is the internal energy (kJ), $\dot{m}$ is the mass flow rate (kg/s), *h* is the specific enthalpy (kJ/kg), ${\dot{Q}}_{CV}$ is the heat transfer rate (kW) and ${\dot{W}}_{CV}$ is the performed power (kW).

When the equipment is a known product, the energy efficiency may be calculated as the ratio of the desired effect to the energy input. For an air conditioning unit, it would be possible to propose an efficiency-based performance coefficient (COP), which is the ratio of the heat removed by the evaporator to the power of the compression. This index is usually higher than unity.

The exergy analysis for a CV can be obtained from Equation (2), where the term *b* is the exergy of a stream (kJ/kg), which is $b=h-h_{0}-T_{0}\left(s-s_{0}\right)$. The exergy efficiency can be calculated by Equation (3), which may be considered as the degradation of the quality of the energy and may be used to assess different types of household appliances [6]. The reference choice was $T_{0}=25$ °C and $P_{0}=100$ kPa

(2) $$\frac{dB_{CV}}{dt}=\sum {\dot{m}}_{e}b_{e}\sum {\dot{m}}_{s}b_{s}+{\dot{Q}}_{CV}\left(1-\frac{T_{0}}{T_{VC}}\right)-{\dot{W}}_{CV}$$

where *B* is the exergy of the control volume (kJ), $\dot{m}$ is the mass flow rate (kg/s), *b* is the specific exergy (kJ/kg), ${\dot{Q}}_{CV}\left(1-T_{0}/T_{VC}\right)$ is the exergy associated with the heat transfer rate (kW) at the surface temperature ($T_{VC}$, in K) and ${\dot{W}}_{CV}$ is the performed power (kW).

A primary difference between the energy and exergy analysis is the Equation (3), which can be applied to any piece of equipment, including refrigeration systems, because these types of thermal machines require modified energy efficiency (COP) to be properly evaluated. In this equation, ${\dot{B}}_{products}$ is the exergy of the products and ${\dot{B}}_{input}$ is the exergy input of the equipment.

(3) $$\eta_{ex}=\frac{{\dot{B}}_{products}}{{\dot{B}}_{input}}$$

**Figure 1:**
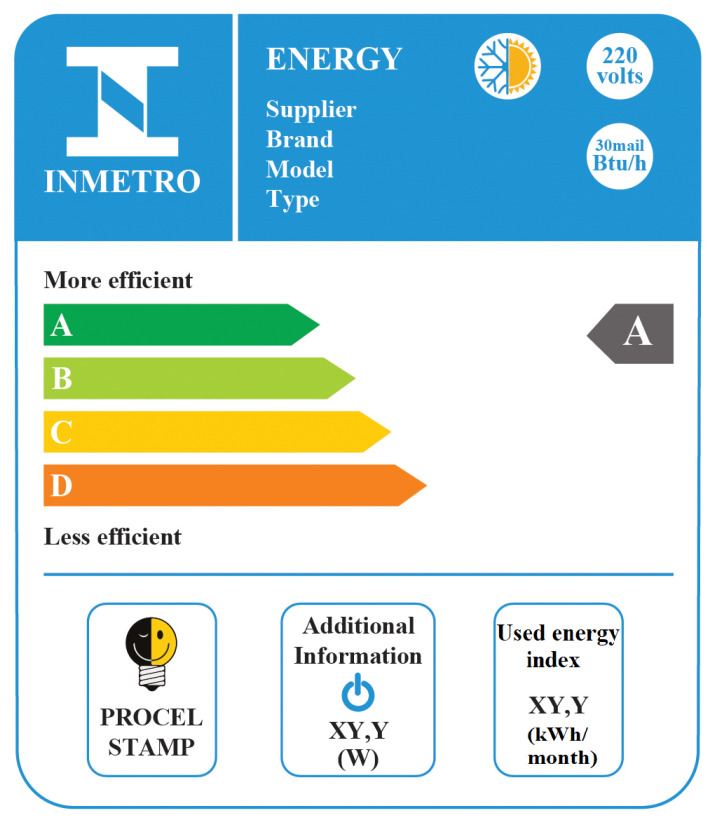
Image of the National Energy Conservation Label, obtained for an air-conditioning unit in INMETRO [26]. This figure was not translated in order to show the unmodified form of label display.

Figure 1 shows a schematic of the National Energy Conservation Label obtained for an air-conditioning unit in INMETRO [26]. For this type of appliance, the objective is to remove energy from the environment (the evaporator) with a certain expenditure of electrical energy (in the compressor of the system). As an example, the model “split high-wall” was chosen. The classes of grade (A being the highest grade) are: (i) A, for COP higher than 3.23; (ii) B, for COP between 3.02 and 3.23; (iii) C, for values between 2.81 and 3.02; and (iv) D, for values between 2.60 and 2.81. The complete information of the label may be seen in the references [24,25].

### 2.1. House Appliances to Heat Water for Showers

**Figure 2:**
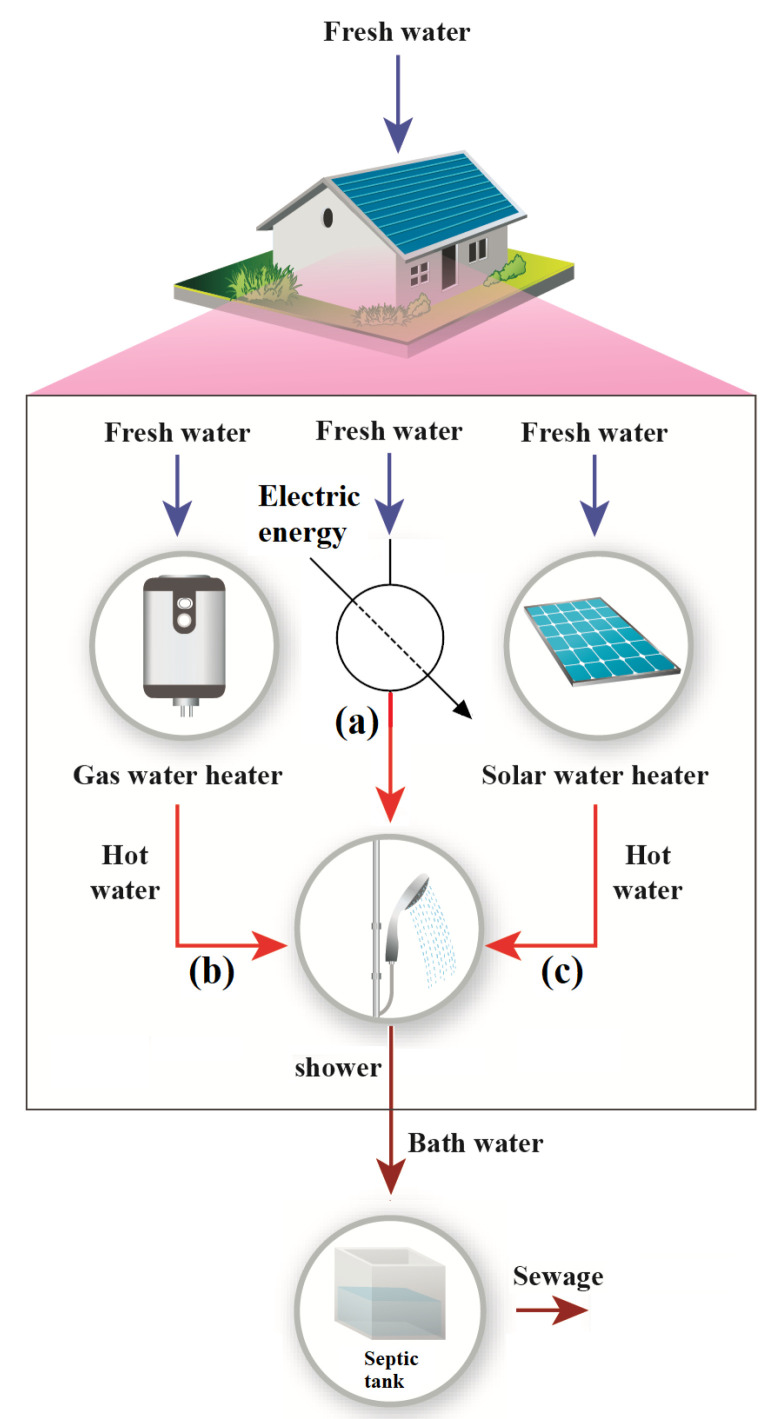
Shower appliances: (**a**) electric shower; (**b**) gas water heater; and (**c**) solar water heater.

Figure 2 indicates that three primary technology methods are utilized in Brazil to heat water. It is essential to highlight that for the sake of the comparison, energy efficiency alone may not be the best tool for discriminating these three methods since the nature of the energy input is different for the same product (water with a given temperature). The electric shower is indicated in (a), gas water heater (b), and solar water heater (c). These technologies are well established in Brazil, nevertheless, electric shower is the most used.

The index used for electric shower labeling is the power consumed, ranging in grade from A to G, since, for all situations, the energy efficiency (Equation (4)) was higher than 95%. The grade “A” was applied for power consumption lower than 2400 W and “G” for power consumption higher than 7900 W [26]. For passage gas water heaters (also known as instantaneous heaters), the energy efficiency is defined by Equation (5). The highest efficiencies were defined as “A” to 84%, and “E” to 76%. For solar water heaters, the index used was the average monthly energy production per square meter (PME), “A” is 80.3 kWh/(month.m${}^{2}$) and “E” 52.3<PME<59.3 kWh/(month.m${}^{2}$). The energy efficiency does not follow the same trend because there are several factors that affect system performance, such as the area and the material. In contrast, the energy produced is a more useful index, since solar energy may be considered as infinite from the perspective of human beings. Nevertheless, nothing is stated regarding the different possibilities of energy use in order to produce similar outcome.

(4) $$\eta_{eletric,shower}=\frac{\Delta {\dot{H}}_{water}}{{\dot{W}}_{input}}$$

(5) $$\eta_{gas,heater}=\frac{\Delta {\dot{H}}_{water}}{{\dot{m}}_{gas}PCI}$$

(6) $$\eta_{solar,heater}=\frac{\Delta {\dot{H}}_{water}}{{\dot{I}}_{solar}A_{collector}}$$

In these equations, the terms $\Delta {\dot{H}}_{water}$ is the enthalpy variation of the water being heated, ${\dot{W}}_{input}$ is the electrical energy, ${\dot{m}}_{gas}PCI$ is the heat of combustion of the natural gas. Also, ${\dot{I}}_{solar}A_{collector}$ is the solar irradiation multiplied by the area of the collector.

With additional information provided in the label [26], for the sake of comparison using the same technology, these previous indexes are satisfactory in helping the consumer to select the most suitable piece of equipment (if the energy input has been decided already). Nevertheless, to compare the energy conversion qualities of these three distinct technologies, it is necessary to use another mechanism. Therefore, the exergy analysis is an analytical tool to assess the quality of the energy conversion process. The destroyed exergy for each appliance can be calculated according to Equations (7)–(9).

(7) $${\dot{B}}_{d,electric}=\dot{W}-\Delta {\dot{B}}_{water}$$

(8) $${\dot{B}}_{d,gas}={\dot{B}}_{gas}-\Delta {\dot{B}}_{water}$$

(9) $${\dot{B}}_{d,solar}={\dot{B}}_{solar}-\Delta {\dot{B}}_{water}$$

In these equations, $B_{d,i}$ is the destroyed exergy of the component *i*, $\dot{W}$ is the power input of the electric shower, ${\dot{B}}_{gas}$ is the chemical exergy of the natural gas [2], ${\dot{B}}_{solar}$ is the exergy associated with the solar radiation [31,32], and $\Delta {\dot{B}}_{water}$ is the exergy variation of the water.

Equations (10)–(12) show the exergy efficiency of the appliances shown in Figure 2, where $\eta_{ex,i}$ is the exergy efficiency of the appliance *i*. These equations show the exergy efficiency, which may be used as a tool to compare different technologies of similar thermodynamic bases (meaning their work performance capability).

(10) $$\eta_{ex,eletric}=\frac{\Delta {\dot{B}}_{water}}{{\dot{W}}_{input}}$$

(11) $$\eta_{ex,gas}=\frac{\Delta {\dot{B}}_{water}}{{\dot{B}}_{gas}}$$

(12) $$\eta_{ex,solar}=\frac{\Delta {\dot{B}}_{water}}{{\dot{B}}_{solar,radiation}}$$

For solar heaters, it was considered that the average solar radiation for an inclined plan was I=0.7 kW/m${}^{2}$ [33] and the sun temperature ($T_{sun}$) was 5777 K.

### 2.2. Energy and Exergy Analysis of Air-Conditioning

**Figure 3:**
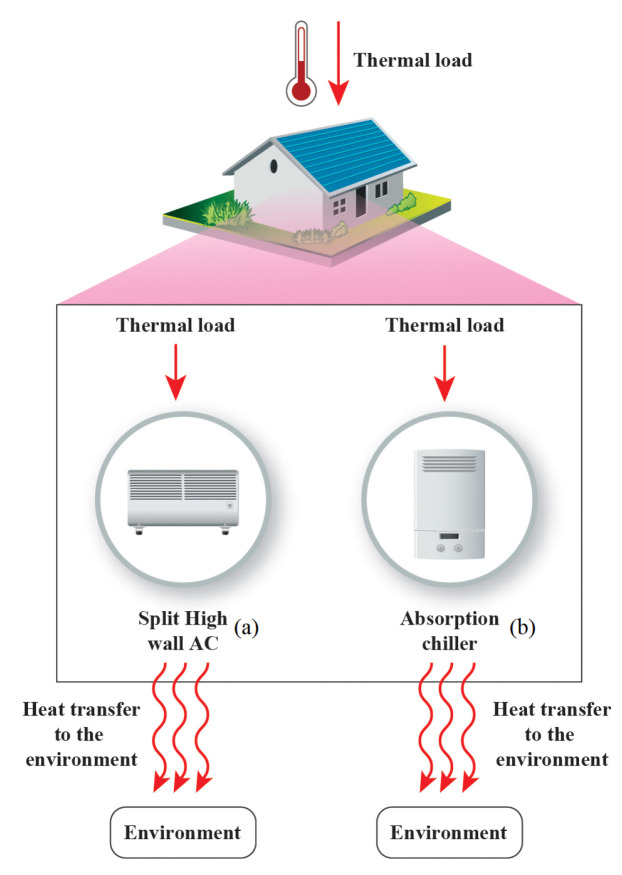
(**a**) Vapor-compression refrigeration cycle. Based on Jabardo and Stoecker [35]; (**b**) absorption cycle using the mixture of water and lithium bromide; based on data of Herold et al. [36].

Another example of the necessity of the second law of thermodynamics is demonstrated in the comparison of two different types of refrigeration cycles. Figure 3 shows a vapor-comparison (a) and an absorption cycle (b). The prime cycle is usually found in appliances of any size from small scale residential to commercial and industrial applications. The absorption refrigeration cycle is usually applied in commercial and most industrial purposes; however, with the increased use of solar thermal heaters, this may also be incorporated into residential systems [34]. For this investigation, both cycles were simulated based on the methods of Jabardo and Stoecker [35] and Herold et al. [36]. The mass, species, and energy balance calculations were carried out in order to determine the value of the same amount of energy removed from the environment, $Q_{evap}=12,000$ Btu/h. Although this value was low for absorption cycles it does represent a common base of comparison in order to demonstrate the applications of the exergy efficiency. It was carried out through a comparison on the same thermodynamics basis, and a modification can be carried out to higher capacities. The exergy analysis was applied for both cycles.

In Figure 3a, $\dot{W}$ is the power delivered to the cycle, and ${\dot{Q}}_{ev}$ is the heat removed from the environment in the evaporation process. For Figure 3b, ${\dot{Q}}_{ge}$, is the energy transferred in the generator to the absorption cycle.

For the vapor-compression refrigeration cycle (Figure 3a), the coefficient of performance (COP) and the exergy efficiency are evaluated according to Equations (13) and (14). It was possible to infer from these equations that both can be used to discriminate between the devices. However, Equation (14) gives the highest value of 100%, whereas the Equation (13) can achieve values of COP higher than unity, considering that it compares heat removed from the environment, ${\dot{Q}}_{evap}$ (lower quality) with electric power, ${\dot{W}}_{comp}$ (higher quality). Being the maximum value, on an energy basis, the Carnot Performance Coefficient (COP). This fact may lead to some misleading information when there is a comparison of different technologies. Nevertheless, since the exergy analysis uses physical quantities of the same nature (maximum available power), e.g., in a heat transfer, there is always an exergy associated to its potential of performing any kind of work. Moreover, if we analyze this useful effect more carefully, it is possible to conclude that the while heat is removed from the environment from the evaporator, the exergy is transferred to the environment (increase the disequilibrium with the reference ambient) as discussed in [37].

(13) $$COP=\frac{{\dot{Q}}_{evap}}{{\dot{W}}_{comp}}$$

(14) $$\eta_{ex,comp}=\frac{{\dot{Q}}_{evap}\left(1-\frac{T_{0}}{T_{evap}}\right)}{{\dot{W}}_{comp}}$$

The absorption conditioning systems (usually available as chiller), Figure 3b, can be evaluated according to its COP (Equation (15)). This value is usually lower compared to the one related to vapor compression cycles, since this type of chiller compares the energy of the same quality (heat transfer rate). As in, it compares the heat provided in the generator with the heat removed from the environment (usually ${\dot{W}}_{pump}$ is smaller than other types of energy transfer).

(15) $$COP=\frac{{\dot{Q}}_{evap}}{{\dot{Q}}_{gen}+{\dot{W}}_{pump}}$$

(16) $$\eta_{ex,abs}=\frac{{\dot{Q}}_{evap}\left(1-\frac{T_{0}}{T_{evap}}\right)}{{\dot{Q}}_{gen}\left(1-\frac{T_{0}}{T_{gen}}\right)+{\dot{W}}_{pump}}$$

Equations (14) and (16) represent the ratio of the same quality (exergy). In the denominator, the quality of the heat in the generator, ${\dot{B}}_{Q_{gen}}={\dot{Q}}_{gen}\left(1-\frac{T_{0}}{T_{gen}}\right)$, and real power is provided in the compressor (${\dot{W}}_{comp}$). Whereas, in the numerator of both equations, there is the exergy transfer associated with the evaporation process, ${\dot{B}}_{Q_{evap}}={\dot{Q}}_{evap}\left(1-\frac{T_{0}}{T_{evap}}\right)$. With exergy efficiency, it was possible to compare two distinct technologies. This index can be used to assess the energy conversion process in a rational manner. Therefore, it may constitute a complementary index to the COP, in order to help the customer decide which technology to invest in.

The two systems were simulated operating at an evaporation temperature ($T_{ev}$) of 3 °C and rejected heat to the environment at 25 °C ($T_{0}$). The temperature of the absorption cycle generator ($T_{ge}$) was set to 80 °C. In order to best compare both cycles, the destroyed exergy was shown as a percentage of the exergy input, e.g., for the compression system, the input was ${\dot{W}}_{comp}$ and the input of energy in the evaporation cycle was ${\dot{Q}}_{gen}$. The terms ${\dot{B}}_{Q_{cond}}$ and ${\dot{B}}_{Q_{abs}}$ were zero, since $T_{cond}=T_{absorber}=T_{0}$.

(17) $${\dot{B}}_{d,comp}={\dot{W}}_{comp}-{\dot{Q}}_{evap}\left(1-\frac{T_{0}}{T_{evap}}\right)$$

(18) $${\dot{B}}_{d,abs}={\dot{Q}}_{gen}\left(1-\frac{T_{0}}{T_{gen}}\right)+{\dot{W}}_{pump}-{\dot{Q}}_{evap}\left(1-\frac{T_{0}}{T_{evap}}\right)$$

## 3. Results and Discussion

### 3.1. Shower Appliances for Heating Water

From the tables of energy consumption and efficiency [26], it was determined that the best, first law, efficiencies of electric showers exceeded 95%, the ones of instant gas heaters approached 84%, and the ones of solar collectors achieved approximately 70%. It is shown from calculations in Table 1 that, by the nature of energy conversion for water heating, all types of equipment were found to have a low exergy efficiency. Moreover, this physical quantity was very similar among all equipments, nearly 3%. Therefore, in all circumstances, 97% of the amount of exergy was destroyed to heat water for a shower. For this particular appliance, the second law efficiency was inexpressive. Therefore, it would be required to further assess the electricity generation efficiency. For example, it would be helpful to determine if it is renewable or non-renewable, proximal or remote from the urban center, or if it has any other distinguishing characteristics [38,39]. From these references, it was found that the energy and exergy efficiency are both needed in order to generate each kW of electricity consumed by the electric shower and for gas flaring (in gas heaters). It is at this stage that solar heaters are distinguished because their input of exergy is almost entirely from renewable sources, as highlighted in Mosquim et al. [5].

Table 1 demonstrates that solar heating exergy efficiency may be considered the best technology to produce hot water. This evaluation takes into account that the surface of buildings is generally not used for any other purpose. The exergy efficiency of solar heating is similar to the other two technologies while the energy efficiency of this appliance is lower than the others. However, because the exergy input is solar, it may be considered as an “infinite renewable source”. The discussion over which type of solar heating may be more efficient, for instance, a comparison between photovoltaic panels and solar water heaters, is out of the scope of this article. As discussed in Pinto and Mady [40], the photovoltaic panels may be more suitable, whereas if there is cogeneration, the solar water heaters may lead to a better efficiency (for the same area).

In Brazil, a high percentage of the energy matrix is based on hydroelectric power (62%) followed by natural gas (13%) [38,39]. Taking into account the way the energy is produced, the data proposed by Florez-Orego et al. [39] on the efficiency of energy-to-electricity conversions such as hydroelectric, 82%, and cogeneration with natural gas, 45%; it is possible to assess a more comprehensive indicator for these appliances. The exergy efficiency of the electric shower becomes 2.1% (hydroelectric) and 1.2% (cogeneration with natural gas). Additionally, knowing that the hydroelectric barrier is generally far from the consumer center, transmission wastes can be considered as 16% [39]. This approach is similar to that performed by Mady et al. [38] in order to assess the impacts of two technologies for power and steam production, yet in the case of caustic soda production, one can correct the values of Table 1. This fact may lead to the conclusion that if there is energy conversion based on natural gas to produce electrical energy, this may also be used in other applications, which is not increasing the temperature of the water, by means of electrical energy, for a shower.

**Figure 4:**
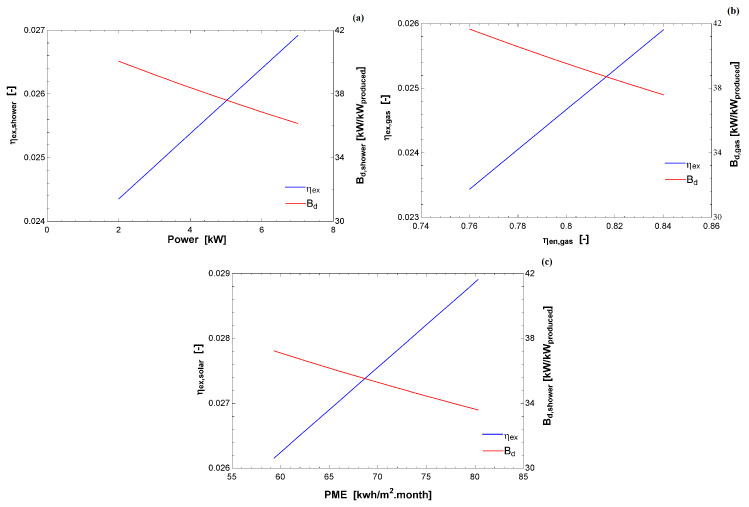
Exergy efficiency (left axis) and destroyed exergy (right axis) and as a function of the energy index used by INMETRO [26]: (**a**) electric shower; (**b**) gas water heater; and (**c**) solar water heater.

Figure 4 indicates the exergy efficiency (in blue) and destroyed exergy relative to the exergy input (in red) as a function of the index used by INMETRO [26]. In all cases, there is a linear correlation with the increase of the energy index with the second law-based calculation. Using the first law, it is possible to compare the same technologies; nevertheless, to fully examine the three different appliances, it was necessary to use the exergy analysis, as indicated by the difference in the abscissa and the similarity in the ordinate axes.

### 3.2. Refrigeration Systems

Table 2 indicates the performance index of two conditioning systems: vapor compression and absorption. The models were solved in EES (Equation Engineering Solver—Fchart) based on information available in Jabardo and Stoecker [35] and Herold et al. [36]. For the vapor compression system, the fluid R410A was chosen, with the specifications found in general technical catalogs. For the absorption system, some conditions of the model were adapted from Herold et al. [36].

It is essential to highlight that the general energy metric indexes, COP, are nearly 3.0 for compression vapor and 0.8 for absorption chillers. The aim of this index is to report the amount of energy removed from the environment (${\dot{Q}}_{evap}$) to the power input (${\dot{W}}_{comp}$). In contrast, the absorption system is the ratio of the ${\dot{Q}}_{evap}$ to the ${\dot{Q}}_{gen}$ (${\dot{W}}_{pump}$ and is negligible. It is noted that the nature of the denominators of these equations is different, based on the following explanation. With the first denominator being power, and the second being heat transfer rate, it is logical that the indexes should not be compared because these are two distinctly different cycles. When the exergy analysis is applied to these systems, the absorption cycle yields higher figures; therefore, this cycle uses the exergy provided more efficiently. It is important to highlight that the results of Table 2 are restricted to one condition ($Q_{evap}=12,000$ btu/h) which may be extrapolated to several thermal loads; nevertheless, the method to compare both cycles is the same and is applicable for residential, commercial, and industrial applications.

**Figure 5:**
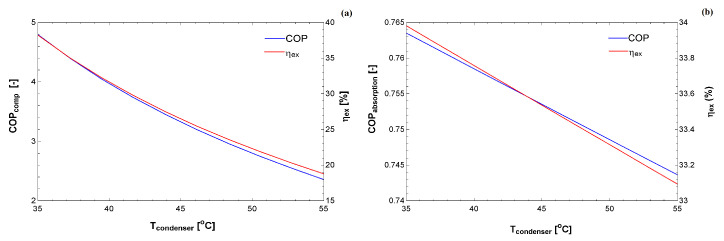
COP and exergy efficiency as a function of the condenser temperature for: (**a**) vapor compression system; and (**b**) absorption system.

**Figure 6:**
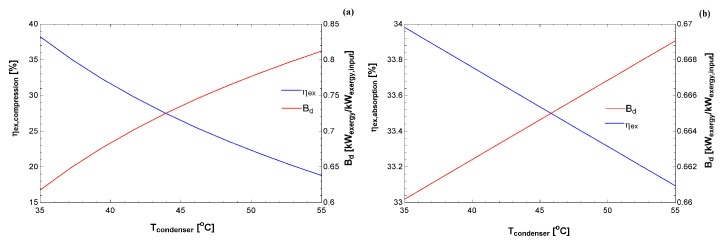
Exergy efficiency and destroyed exergy (relative to the exergy input) as a function of the condenser temperature for: (**a**) vapor compression system; and (**b**) absorption system.

Figure 5 and Figure 6 compare the exergy efficiency of the two cycles for different temperatures at the condenser. As expected, the performance coefficient decreased as a function of the condensation temperature. The exergy efficiency also decreased, although this provides a clue for each condition as to which cycle uses the exergy better. It is essential to bear in mind that these simulations were carried out for specific requirements. It is essential that the figures in the exergy basis are in the same order of magnitude, whereas the COP of the compression cycle is higher than the absorption cycle.

Figure 6 indicates the exergy destroyed rate (normalized by the exergy input) for both cycles. Interestingly, the generalized destroyed exergy is lower for this absorption cycle, indicating that, from the exergy input, a smaller percentage was destroyed compared to the compression cycle. Therefore, these results register additional information from the first law of thermodynamics. Where the exergy parameters may be used to compare different technologies for the same purpose (increase the temperature of the water, cool some environment, and other appliances). A careful analysis should be carried out to indicate which cycle is better as a function of the thermal load of the environment. From these records, public policies may be introduced to investigate the usage of equipment that leads to lower environmental impacts, which in turn would lead to an economic gain for consumers who buy these technologies.

### 3.3. Final Considerations of the Exergy Index

As demonstrated in the previous analyses the exergy analysis may be used as a tool to compare different technologies, and it should be used as an complementary index to the actual energy indexes used in “INMETRO Brazilian Labeling Program”.

The exergy efficiency should follow a similar trend from the energy indexes, and a reference of the “quality of the energy transfer” may be added to the label. From A to G (as in the actual program).

## 4. Conclusions

In this article, a simple idea was proposed in the Brazilian context to complement the labels of Procel and the National Energy Conservation Label [26] programs that were designed based on principles of the first law of thermodynamics. The purpose was to incorporate the usage of the second law of thermodynamics to help increase the capability of the consumer to discern which technology may have a higher exergy demand and environmental impact.

This investigation found that the exergy analysis provided the true losses of an energy conversion process and therefore has the potential to be used as a tool to quantify multiple methods for obtaining a similar outcome. It was demonstrated from the exergy analysis that the indexes converged into proximity values, around 3%.

For refrigeration systems, it was demonstrated that the exergy analysis can be used to carry out comparisons for residential, commercial, and industrial appliances. In this experiment, a low thermal load (12,000 btu/h) was used to compare residential appliances (even if the absorption cycle did meet this demand); nevertheless, a basis of thermodynamic investigation is proposed for carrying out future reports. Moreover, it may be preferable to use absorption refrigeration cycles depending of its initial costs. A more detailed analysis may be carried out, since the price of split-highwall is very attractive, and the demand of the absorption cycle must be higher than those indicated in this article.

Complimentary future research that may be considered might include investigations into how humans use natural resources and how the changes in our habits and public policies may be a basis from which we can achieve a more sustainable society as outlined by [29]. Therefore, public policies may be a basis for the way energy and natural resources are used in the nation, bearing in mind that the use of the electrical shower accounts for 30% of electricity consumption in Brazilian households [24].

The reason for this article is to propose an additional tool for policies-making and decision buyers. That the quality of the energy may lead to better resource usage, and even a fast payback time, based on the second law of thermodynamics. To understand the consumption of electrical shower, most citizens may compare with the number of lamps, for instance. Yet, this is a case study in Brazil, with its own realities and differences in education. Therefore, the information A to G of quality of energy use would be adequate to facilitate the consumer.

## Figures and Tables

**Table 1:** Comparative energy and exergy analysis of studies of the three technologies: electric shower, gas water heater and solar heater; for a temperature increase in the system of 20 °C from the environmental conditions $T_{0}$ = 25 °C; in this table, only the best pieces of equipment were analyzed.

Equipment	Variable (Ref. [26])	Energy Index	Equation	$\eta_{\mathrm{ex}}$
Electric shower	Power	7 kW	(10)	2.6%
Gas water heater	$\eta_{en}$	84%	(11)	2.6%
Solar heater	PME	80.3 kWh/(month.m${}^{2}$)	(12)	2.7%

**Table 2:** Comparative energy and exergy analyses of conditioning systems, for fluid temperature leaving the condenser at 45 °C.

-	Equation	First Law Metrics	Equation	Second Law Metrics
Vapor comp. system	(13)	COP=3.1	(14)	$\eta_{ex}=27.3$%
Absorption system	(15)	COP=0.75	(16)	$\eta_{ex}=33.5$%
